# 

**Published:** 1838-07-01

**Authors:** 


### THE MEDICO-CHIRURGICAL REVIEW,

AND

### JOURNAL

OF

### PRACTICAL MEDICINE.

*(NEW SERIES.)*

VOLUME TWENTY-NINE,

[1st of APRIL to 30th of SEPTEMBER,]

1838.

VOL. IX. of DECENNIAL SERIES.

EDITED

BY JAMES JOHNSON, M.D.

PHYSICIAN EXTRAORDINARY TO THE LATE KING,

AND

HENRY JAMES JOHNSON, Esq.

LECTURER ON ANATOMY AT THE SCHOOL OF ST. GEORGE'S HOSPITAL IN KINNERTON STREET.

Surgeon Genl's Office LIBRARY Washington, D.C. 57469 brown

LONDON:

PUBLISHED BY S. HIGHLEY, 32, FLEET STREET,

*Re-printed in New York, by Mr. Wood.*

1838.

WI
ME 75 T

PRINTED BY F. HAYDEN,
Little College Street, Westminster.

### BIBLIOGRAPHICAL RECORD.

1. The Substance of Two Lectures upon the Arguments to be derived from the Physical Organization and Moral Constitution of Man, in proof of his Religious Obligations. By WILLIAM BREWER, M.D. 8vo, pp. 54. Highley, 1838.

☞ *A very good medico-theological discourse, well adapted for a popular audience.*

---

2. An Essay on Children, and that Disease designated Mesenteric Obstruction, Atrophy, or Marasmus, adapted partly for Parents, &c. By A. L. PEARCE, Surgeon. Duodecimo, pp. 225. Sherwood and Co. 1838.

---

3. A History of British Reptiles. By THOMAS BELL, F.R.S. Illustrated by Wood-cuts of each Species. Part I. price 2s. 6d. Van Voorst, Paternoster Row. 1838.

---

4. On the Reform of Criminals and Prisons. Octavo, pp. 20. Sewed. V. and R. Stevens, Bell-yard, Lincoln's-Inn.

---

5. Regulations and Instructions for the Medical Officers of His Majesty's Fleet. London, 1835.

☞ *We have noticed these excellent instructions in another department of the Journal.*

---

6. The Medical Examiner. Nos. 1, 2, 3, 4. Jan. 3d to Feb. 14th, 1838. Edited by Drs. BIDDLE and CLYMER. Published twice a month in Philadelphia, by J. G. Anner. Each number contains 16 pages, in double columns.

☞ *We have noticed this new periodical in the body of the Journal.*

---

7. Velpeau's Anatomy of Regions. Translated from the French. By HENRY HANCOCK, Lecturer on Practical and Surgical Anatomy at the Westminster Hospital School, &c. Octavo, pp. 570. Longman and Co. 1838.

---

8. Medical Portrait Gallery. Biographical Memoirs of the most celebrated Physicians, Surgeons, &c. who have contributed to the advancement of Medical Science. By T. J. PETTIGREW, F.R.S. &c. Part the Second, price 3s. To be continued monthly.

☞ *This part contains portraits and memoirs of Ruysch, Haller, and Sir A. Carlisle.*

---

9. Elementa Praxeos Medicæ; or a Manual of the Practice of Medicine, for the Use of Students and Junior Practitioners. By D. SPILLAN, M.D. 32mo. Renshaw. Price one Shilling. 1838.

---

10. Dr. SPILLAN's Medical Formulæ, 32mo. Price one Shilling. Renshaw, 1838.

---

11. A Practical Treatise on Fractures. Illustrated with Sixty Wood-cuts. By EDWARD F. LONSDALE, Surgeon, Demonstrator of Anatomy at the Middlesex Hospital School of Medicine, &c. Octavo, pp. 536. Churchill, London, 1838.

---

12. Guy's Hospital Reports, No. VI. April, 1838. Highley, London.

---

13. Hints on Animal Magnetism, addressed to the Medical Profession in Great Britain. By J. C. COLQUHOUN, Esq. Advocate. Octavo, pp. 48. Ed. 1838.

---

14. The Nature and Treatment of Diseases of the Ear. By Dr. WILLIAM KRAMER. Translated from the Second Edition of the German, with the latest Improvements of the Author. By JAMES RESDON BENNETT, M.D. Octavo, pp. 306. Longman & Co. Longdon, 1838.

---

15. On the Treatment of Cataract without Operation, at the Royal Ophthalmic Hospital, Moorfields, under the Auspices of Mr. Tyrrell. By LOUIS FRANCOIS GONDRET, M.D. Highley, Fleet Street, 1838.

---

16. The Medico-Chirurgical Pharmacopœia; or a Conspectus of the best prescriptions in Medicine, Surgery, Obstetricy, and Infantile Medicine; with a Table of Doses of all Medicines in use—the Additions in the London Pharmacopœia, &c. &c. By MICHAEL RYAN, M.D. &c. Lecturer on Medicine, &c. Duodecimo, pp. 327. Churchill, London, 1838.

☞ *There is a vast mass of information collected into close compass in this little work. Though some of the subjects do not appear to harmonize with one another, yet*

*they are all useful at the bedside of sickness, or in the short hour of leisure from professional toils and anxieties.*

17. Remarks on Dr. Handyside's Case of Suicide. By JOHN ROSE CORMACK, M.D. Pp. 16. Carfrae, Ed. 1838.

18. A Manual of British Botany; in which the Orders and Genera are arranged and described according to the natural System of DE CANDOLLE; with a Series of Analytical Tables for the Assistance of the Student in the Examination of the Plants indigenous to Great Britain, &c. By D. MACREIGHT, M.D., F.R.C.P. Lecturer on Materia Medica and Therapeutics, &c. &c. Octavo, pp. 206. Churchill, April, 1838. Price 7s. 6d.

☞ *There is a prodigious mass of elementary matter and useful information in this pocket volume.*

19. Aphorisms on natural and difficult Labours, Puerperal Diseases, and on the Management of Infants. (SECOND EDITION.) By MICHAEL RYAN, M.D. In a duodecimo, pp. 128, with copious Index. Churchill, 1838, price 2s.

☞ *Dr. Ryan observes that "this little work is arranged as a pocket companion for the use of students and junior practitioners. It is only intended for the obstetric chamber, and not for the minute study of the important branch of surgery of which it treats." In this respect it is certainly a valuable* VADE MECUM.

20. The India Journal of Medical and Physical Science. Edited by F. CORBYN, Esq. Nos. IX. X. XI. and XII. Calcutta, 1837.

21. The India Review, and Journal of Foreign Science and the Arts. Edited by FRED. CORBYN, Esq. Nos. 18, 19, & 20. Calcutta, 1837.

22. A Letter addressed to the Gentlemen of the Medical Profession on the Nature and Properties of the Aluminous Chalybeate Water at Sand Rocks, in the Isle of Wight. By T. L. WATERWORTH, Surgeon. Octavo, sewed, pp. 28. Newport, 1838.

23. Experiments and Observations on the Gastric Juice and the Physiology of Digestion. By W. BEAUMONT, M.D. Reprinted from the Plattsburgh Edition, with Notes. By AND. COOMBE, M.D. Edinb. 1838. Pp. 319. Price 7s.

24. The Quarterly Journal of the Calcutta Medical and Physical Society. Nos. 3 and 4. Edited by Drs. GOODEVE and O'SHAUGHNESSEY. July and December, 1837.——*In exchange.*

25. A Series of Anatomical Sketches and Diagrams, with Descriptions and References. By THOMAS WORMALD, Assistant Surgeon and Demonstrator of Anatomy at St. Bartholomew's Hospital, and ANDREW MELVILLE M'WHINNIE, Teacher of Practical Anatomy at Bartholomew's Hospital. Part I. Price 4s. Quarto, pp. 16, with 5 plates. Highley, April, 1838.

26. A History of British Birds. By W. YARRELL, F.L.S. Illustrated by a Woodcut of each Species, and numerous Vignettes. Part VI. price 2s. 6d. Van Voorst, Paternoster Row, May 1, 1838.

☞ *This is a remarkably good part.*

27. Buxton and its Waters; an Analytical Account of their Medicinal Properties and General Effects. By W. H. ROBERTSON, M.D. Physician to the Buxton Bath Charity. Duodecimo, pp. 147. Charles Tilt, Fleet Street, 1838.

28. A Series of Anatomical Plates, in lithography, &c. By JONES QUAIN, M.D., and W. J. ERASMUS WILSON. Fasciculi 47-8-9. Price 2s. each. March, April, May, 1838.

29. Phrenology Vindicated, in a series of Remarks, Physiological, Moral, and Critical, on Article VII of the November number of the "CHRISTIAN Examiner," headed "Pretensions of Phrenology examined." By CHARLES CALDWELL, M.D. 8vo. pp. 93. Lexington.

☞ *This is the most lacerating scourge that has ever been applied to the backs of anti-phrenological bigots, fanatics, and hypocrites.*

30. Remarks on the Influence of Mental Cultivation and Mental Excitement upon Health. By AMARIAH BRIGHAM, M. D. Second Edition. Boston, 1833.

31. Observations on the Influence of Religion upon Health and Physical Welfare of Mankind. By AMARIAH BRIGHAM, M.D. Boston, 1835.

*** *We are sorry we did not receive these little works sooner; but we hope to notice at least the latter of them in our next number.*

32. The Article on Insanity in the 94th Number of the North American Review, for January 1837. By the REVIEWER.

*** *This is a very clever article, and we suspect it is from the pen of Dr. Brigham.*

33. On the Improvement of Medicine. The Oration delivered before the Medical Society of London, March 8, 1838. By THEOPHILUS THOMPSON, M.D.

*** *A very creditable production.*

34. Outlines of Human Physiology; designed for the use of the higher classes in common schools. By GEORGE HAYWARD, M. D. Professor of Surgery and Clinical Surgery in Harvard University.

*** *This essay is conveyed in simple language and is well adapted for the readers to whom it is addressed.*

35. On the Use and Abuse of the Pessary. By J. T. SHARPLESS, M.D. of Philadelphia. 8vo. pp. 12.

36. The Medical Examiner. Nos. 1 to 9 inclusive. Philadelphia 1838. *In exchange.*

*** *Liverpool will be an inconvenient channel. Packets are put into the Post Office there, and come to town with from* 3 *to* 10 *pounds sterling postage! We are consequently obliged to refuse them. The Examiner came thus, but, from courtesy, was afterwards delivered to us free of expense. We shall reciprocate through a private channel, the first opportunity.*

37. A Practical Compendium of the Materia Medica; with numerous formulæ adapted to the treatment of the Diseases of Infancy and Childhood. By ALEX. URE, M.D. M.R.C.S. London. Duodecimo, pp. 240. Schloss, Great Russell Street, price 6s.

*** *There is much novelty in this plan, which is taken from a German model (Frankel's,) but embodies a large mass of useful and practical observations from some of our best Authors.*

38. The Physiognomy of Mental Diseases. By ALEX. MORRISON, M.D. Nos. 1 and 2, with plates, price 3s. 6d. each. Highley, 1838.

*** *This is a work which cannot be reviewed. But it is worthy of praise.*

39. A Dictionary of Practical Medicine, &c. By JAMES COPLAND, M.D. Part 5, price four shillings. Longmans, 1838.

40. Practical and Experimental Chemistry, adapted to Arts and Manufactures. By E. MITSCHERLICH, Professor of Chemistry in the University of Berlin. Translated from the first portion of his Compendium. By STEPHEN LOVE HAMMICK, M. D., Fellow of the Royal College of Physicians, and one of the Radcliffe Travelling Fellows of the University of Oxford. 12mo. pp. 316, London, 1838.

*** *Dr. Hammick, the translator of Mitscherlich, is a gentleman of considerable attainments, and well qualified in every way to do justice to his author. He has taken great pains with his translation, and it is remarkably well executed. We recommend the work to the lovers of practical chemistry. It will be a valuable addition to the library and the laboratory.*

41. Des Maladies Mentales, considerées sous le Rapports Medical, Hygienique, et Medico-legal. Par E. ESQUIROL. Accompagnées de 27 Planches Gravés. Deux Tomes, 8vo. pp. 678—864. Paris. 1838.

*** *In our next.*

42. An Introduction to the Study of Animal Magnetism. By the Baron DU-POTET DE SENNEVOY. With an Appendix containing Reports of British Practitioners in favour of the Science. 12mo. pp. 388. London, 1838.

